# The Role of Intra-Operative Duplex Ultrasonography Following Translabyrinthine Approach for Vestibular Schwannoma

**DOI:** 10.3389/fsurg.2022.853704

**Published:** 2022-04-28

**Authors:** Nida Fatima, Zachary R. Barnard, Anne K. Maxwell, Tommy J. Muelleman, William H. Slattery, Gautam U. Mehta, Willis Wagner, Gregory P. Lekovic

**Affiliations:** ^1^ Division of Neurosurgery, House Institute, Los Angeles, California, United States; ^2^ Department of Otolaryngology, House Institute, Los Angeles, California, United States; ^3^ Department of Neurosurgery, Cedars-Sinai Medical Center, California, United States

**Keywords:** vestibular schwannoma, tranlabyrinthine craniotomy, ultrasound, Sigmoid sinus, Sinus occlusion

## Abstract

**Objective:**

Sigmoid sinus (SS) stenosis is a complication of translabyrinthine approach. Velocity changes in the SS measured by intra-operative doppler ultrasound may help in identifying patients at risk for sinus occlusion.

**Patients:**

SS velocity was measured using doppler ultrasound prior to opening dura and again prior to placement of the abdominal fat graft.

**Intervention:**

Data collected included: patient age, surgical side, sinus dominance, tumor volume, intra-operative doppler ultrasound measurements, post-operative venous sinus imaging, anticoagulation, and morbidities and mortalities.

**Main Outcome Measure:**

SS patency and velocity.

**Results:**

Eight patients were included in the analysis (22 to 69 years). Four had left-sided and four had right-sided craniotomies. Sigmoid sinuses were either right-side dominant or co-dominant. The mean velocity ± standard deviation (SD) prior to dura opening and abdominal fat packing was 23.2 ± 11.3 and 25.5 ± 13.9 cm/s, respectively, *p* = 0.575. Post-operative Magnetic Resonance Venography (MRV) imaging showed four sigmoid sinus occlusions; seven patients showed sigmoid sinus stenosis, and one internal jugular vein occlusion. One patient had post-operative Computed Tomography Venography (CTV) only. Of the four patients with MRV occlusions, CTVs were performed with three showing occlusion and all four-showing stenosis. One patient with internal jugular vein occlusion on MRV received warfarin anticoagulation. There was one cerebrospinal fluid leak requiring ear closure, one small cerebellar infarct, and one with facial nerve palsy (House-Brackman Grade 3).

**Conclusion:**

SS velocity changes before and after tumor resection were not predictive of sinus occlusion. We hypothesize that sinus occlusion may be caused by related factors other than thrombosis, such as external compression of the sinus secondary to abdominal fat grafting.

## Introduction

Sigmoid sinus (SS) stenosis and/or occlusion is a known complication of the translabyrinthine approach (TLC) for vestibular schwannomas. The etiology of stenosis/occlusion may be presumed to be due to thrombosis of the sinus. This may be recognized during surgery such as when the sinus is packed due to injury during exposure or unrecognized, such as thrombosis occurring secondary to thermal injury during drilling or during prolonged exposure to heat from the operative microscope. The risk of propagation of clot from a thrombosed sinus may in turn lead to therapeutic anticoagulation. There have been a number of retrospective analysis looking at sinus thrombosis diagnosis and treatment strategies although none have yielded a consensus (1–5).

The goal of the present study was to determine whether sinus occlusion could be recognized intra-operatively with the use of duplex ultrasonography of the exposed sigmoid sinus. To our knowledge, there are no studies that have evaluated intra-operative flow of the SS during a TLC using doppler ultrasonography. This technique may yield an etiology or potential intervention for SS thrombosis or compression. We describe the technique of using intra-operative doppler ultrasound as a method for assessing velocity changes equating to narrowing of the SS. We believe that this may aid in changing operative technique as well as directing post-operative management including, obtaining venous sinus imaging or the need for medical therapy.

## Methods

This study was approved by the institutional review board at St. Vincent’s Hospital (IRB# SV-19-026). The data from all consecutive patients undergoing translabyrinthine approach during the study period were prospectively gathered and analyzed.

All patients underwent a standard translabyrinthine approach for resection of vestibular schwannomas. In brief, this procedure was performed as follows: a semicircular incision was made with separate scalp and T-shaped muscular flaps. Mastoidectomy, labyrinthectomy, and drilling of the internal auditory canal (IAC) were performed with round cutting and diamond burrs of various sizes. The bone posterior to the SS was drilled in order to completely skeletonize the SS in order to allow for optimal operative corridor, including intermittent compression of the sinus during dissection. If present, any remaining layer of bone overlying the SS following translabyrinthine exposure (aka “Bill’s Island”) was removed prior to dural opening. The dura was then opened, retracted posteriorly, and tumor resection proceeded in the usual fashion. An abdominal fat graft was harvested and woven into the dural opening. The site was then covered with a titanium mesh cranioplasty followed by layered closer of the muscle, dermis, and skin.

**Figure 1:**
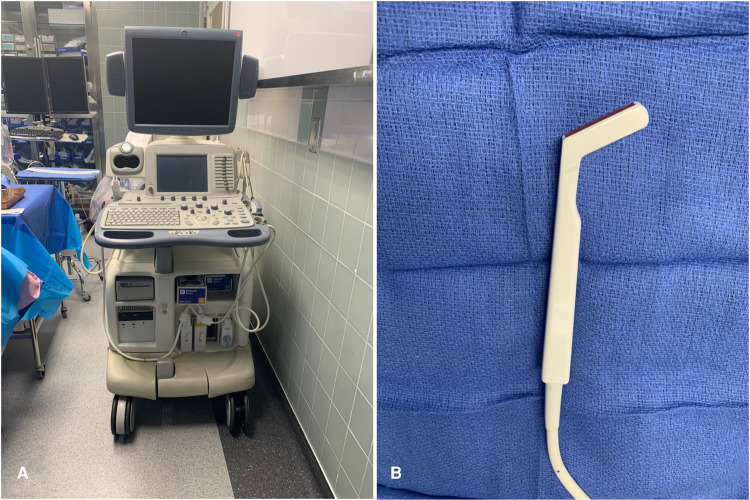
(**A**) Intra-operative GE ultrasound used for measuring sigmoid sinus velocities. (**B**) The hockey stick probe used for measuring sigmoid sinus velocities.

**Figure 2:**
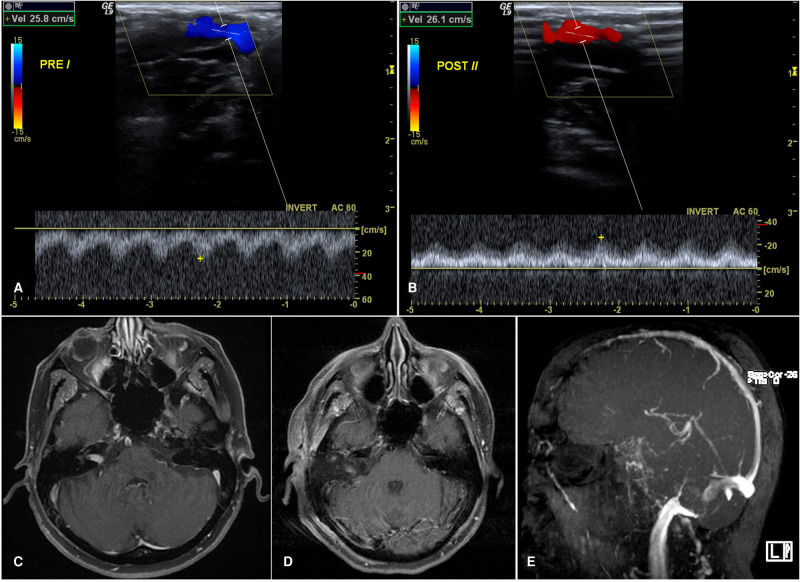
69 year-old female with a right intracanalicular vestibular schwannoma who underwent a translabyrinthine approach for resection due to worsening of clinical symptoms. (**A**) Doppler ultrasound of the sigmoid sinus prior to dural opening. (**B**) Doppler ultrasound of the sigmoid sinus prior to closure. (**C**) Pre-operative MRI brain with contrast demonstrating an intracanalicular vestibular schwannoma. (**D**) Post-operative MRI with contrast and fat saturation showing a gross total resection of the vestibular schwannoma. (**E**) Post-operative MRA-TOF of the brain with significant stenosis of the right sigmoid sinus.

Ultrasonographic evaluation of the SS including velocity measurement was performed at two timepoints during surgery: before opening the dura and after tumor resection, prior to placement of the abdominal fat graft. A GE Logiq ultrasound (General Electric, Boston, MA) in M-mode with the hockey stick probe (**Figure 1A,B**) was used for measurement of the SS velocity along with 2D imaging. The venous sinus ideal waveform was identified and a still picture taken. The velocity (cm/s) of blood flow was measured as the peak amplitude (**Figure 2A,B**). Three measurements were taken from the inferior, middle, and superior segments of the SS at each time point and used for statistical analysis. Statistical analysis was performed using Wilcoxon signed rank test (IBM SPSS Statistics 24).

## Results

Eight patients undergoing a translabyrinthine approach for vestibular schwannoma removal were underwent intra-operative doppler ultrasound measurements and associated post-operative SS imaging (**Table 1**). Ages ranged from 22 to 69 years old. There were four left-sided and four right-sided craniotomies. Sigmoid sinuses were either right-sided dominant or co-dominant.

**Table 1:** Patient demographics and results.

Patient	Age at Surgery (yrs)	Surgery Side	Sinus Dominance	MRV SS Occluded	MRV SS Compression	MRV IJ Occluded	CTV SS Occluded	CTV SS Compression	CTV IJ Occluded	Pre-dural opening mean velocity (cm/s)±SD	Pre-closure mean velocity (cm/s)±SD	Tumor Volume (cm^3^)	Complications
1	55	Left	Co-dominant	Yes	Yes	No	Yes	Yes	No	24.23 ± 2.97	21.63 ± 3.48	1.482	HB 3
2	62	Right	Co-dominant	Yes	Yes	No	Not Done	Not Done	Not Done	29.27 ± 3.56	27.03 ± 3.52	5.75	None
3	22	Left	Co-dominant	No	Yes	No	Not Done	Not Done	Not Done	44.13 ± 4.05	44.3 ± 11.97	38.94	None
4	51	Left	Right	Not Done	Not Done	Not Done	Yes	Yes	No	18.23 ± 0.58	10.33 ± 0.91	25.585	Left cerebellar infarct
5	56	Left	Right	Yes	Yes	Yes	Yes	Yes	No	6.53 ± 0.55	9.17 ± 1.60	7.728	None
6	69	Right	Co-dominant	No	Yes	No	Not Done	Not Done	Not Done	22.80 ± 2.08	23.73 ± 3.84	0.08	None
7	57	Right	Right	No	Yes	No	Not Done	Not Done	Not Done	12.97 ± 1.53	20.60 ± 6.37	9.72	None
8	63	Right	Right	Yes	Yes	No	No	Yes	No	30.83 ± 4.86	46.87 ± 3.54	0.729	CSF leak–> ear closure

The mean velocity ±SD prior to dura opening and abdominal fat packing was 23.2 ± 11.3 and 25.5 ± 13.9 cm/s, respectively, *p* = 0.575. The mean post-operative MRV was done in 1.5 months (range, 0.3–5 months). Post-operative MRV imaging showed four patients with sigmoid sinus occlusions. All eight patients had sigmoid sinus compression and one had an internal jugular vein occlusion. One patient had only a post-operative CTV due to the inability to get an MRI. On the four patients with MRV occlusions, confirmatory contrast enhanced CTVs were obtained with no discrepant findings. The one patient with internal jugular vein occlusion on MRV received warfarin anticoagulation.

MRI T1 showing the fat packing in patients with no SS stenosis (A) and stenosis (B) in the early postoperative period.

Complications included one CSF leak requiring ear closure, one small cerebellar infarct and one facial nerve palsy (House-Brackman grade 3). None of the patient has any symptomatic complications except the patient with facial nerve palsy which improved gradually.

## Discussion

Sigmoid sinus (SS) stenosis and/or occlusion is a known complication surgery for vestibular schwannomas (1). Post-operative non-invasive imaging (either MRV or CTV) has been used to assess sigmoid sinus patency when compromise is suspected (4, 6), such as in patients complaining of symptoms of elevated intracranial pressure including headache and CSF leak. Recent studies have shown that the incidence of post-operative stenosis of the lateral sinus following translabyrinthine surgery may be higher than previously thought (2). The clinical significance of asymptomatic post-operative stenosis of sigmoid sinus following lateral skull base surgery, however, is not established, especially with regard to whether or not prophylactic anti-coagulation is required in the presence of an asymptomatic stenosis (1, 2). In our opinion, the need for anti-coagulation also depends on the presumptive etiology of the stenosis: stenosis, or even occlusion, due to external compression of the sinus can be treated more expectantly as there is presumably little or no risk of clot propagation within the sinus, whereas thrombosis caused by damaged endothelium may result in a higher risk of morbidity.

Vascular complications including arterial and venous infarcts, venous sinus occlusion, and hematomas are known to occur after skull base surgery and can be a major source of morbidity and mortality. The identification and etiology of SS occlusion and compression are necessary to help mitigate associated morbidity. Post-operative imaging has classically been used to assess sigmoid sinus patency when compromise is suspected. Unfortunately, this does not allow for real-time monitoring and potential intervention. We conducted intra-operative measurements of sigmoid sinus velocity before and after opening the dura in order to evaluate whether intraoperative thrombosis of the sinus could be reliably detected, and whether thrombosis was the etiology of post-operative stenosis. By measuring the velocity of the sigmoid sinus at multiple time points throughout the surgical case we may better understand the possible cause of sinus occlusion or compression. Etiology has been linked to multiple intra-operative techniques such as thermal injury from the operating microscope and high-speed drills, electrocautery, and fat graft compression. Although avoiding these may be difficult there are maneuvers that may diminish risk such as water cooling a drill bit, reducing the intensity of the microscope light, limiting electrocautery on the sinus, and avoiding overpacking the approach site with abdominal fat.

We did not see any differences between the pre-dural opening and the pre-closure SS velocities (**Figure 2A,B**). However, it was not powered to detect a difference between the two groups only to establish the feasibility of using doppler ultrasound to measure velocities. In line with this goal, our measured velocities of the sigmoid sinus aligned with a prior study that measured the SS velocity with endovascular techniques. They found a median normal SS velocity range of 10–67(cm/s) and SS velocity range of 49–182 (cm/s) in sinus stenosis (7).

**Figure 3:**
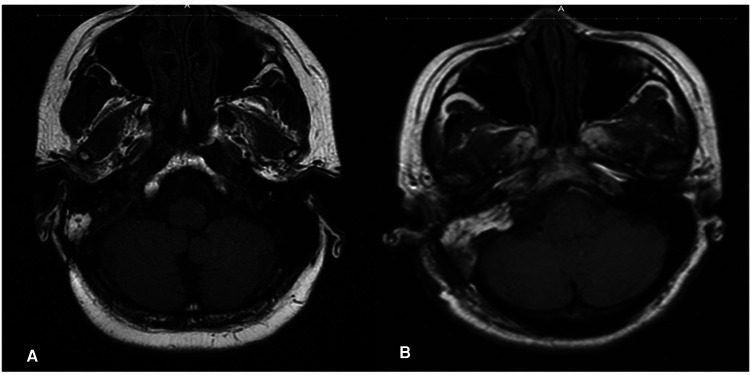
MRI T1 showing the fat packing in the patient with no stenosis (**A**) and stenosis (**B**) in the early postoperative period.

Interestingly, there were no major changes in the velocity between our two measured time points in spite of the post-operative imaging demonstrating significant stenosis and/or occlusion of the sinus. Were the sinus stenosis to have been caused by intra-operative thrombosis, we could expect to see increases in the pre-closure measurements indicating a narrowing of the SS. This was not observed. Rather, our data support the conclusion that stenosis of the sinus occurs after our last ultrasound measurement; therefore, it is only appreciated on the post-operative imaging (**Figure 2C,D**). We believe the most likely explanation of our results is that stenosis of the sinus was seen following compression of the sinus from excessive packing of the fat graft and subsequent mesh cranioplasty. Importantly, after placement of the mesh cranioplasty the ability to use doppler ultrasound is limited (**Figure 3A,B**).

Limitations: Our study is limited due to, (i) small sample size, (ii) retrospective in nature, (iii) one study center. However, we believe the data is sufficient to conclude that SS velocity is not predictive of SS stenosis on non-invasive post-operative imaging. Further studies with larger numbers of patients are necessary to assess the sensitivity of Doppler ultrasound in the diagnosis intra-operative sinus thrombosis, and/or predict post-operative stenosis. Finally, clinicians deciding whether therapeutic anti-coagulation is indicated for post-operative SS stenosis or occlusion may need to take into consideration that causes other than sinus thrombosis, such as abdominal fat grafting and titanium mesh cranioplasty, may contribute to post-operative stenosis seen on non-invasive post-operative vascular imaging.

## Conclusion

Intra-operative duplex ultrasound is a novel technique for assessing SS patency during translabyrinthine approach. Using intra-operative duplex ultrasound may provide immediate information regarding thrombosis of the SS which may in turn assist in intra- and post-operative management.

## Data Availability

The original contributions presented in the study are included in the article/supplementary material, further inquiries can be directed to the corresponding author/s.
